# Microvascular changes in children with MIS-C: a monocentric exploratory study using comparative nailfold capillaroscopy

**DOI:** 10.3389/fped.2025.1678064

**Published:** 2025-10-14

**Authors:** Sema Nur Taşkın, Şeyda Doğantan, Esra Esen, Pınar Garipçin Sarı, Benhur Şirvan Çetin, Ayşenur Paç Kısaarslan, Muammer Hakan Poyrazoğlu

**Affiliations:** ^1^ Division of Pediatric Rheumatology, Department of Pediatrics, Erciyes University Faculty of Medicine, Kayseri, Türkiye; ^2^ Division of Pediatric Infectious Diseases, Department of Pediatrics, Erciyes University Faculty of Medicine, Kayseri, Türkiye

**Keywords:** MIS-C, COVID-19, children, nailfold capillaroscopy, microvascular damage, endothelial dysfunction

## Abstract

**Background:**

Multisystem Inflammatory Syndrome in Children (MIS-C) is a severe hyperinflammatory condition that arises after SARS-CoV-2 infection and may lead to endothelial dysfunction and microvascular damage. Despite increasing knowledge on systemic manifestations, microcirculatory involvement in MIS-C remains underexplored.

**Objective:**

To evaluate nailfold capillaroscopy (NFC) findings in children diagnosed with MIS-C and compare them with age- and sex-matched healthy controls, thereby assessing subclinical microvascular alterations associated with MIS-C.

**Methods:**

In this cross-sectional study, 25 MIS-C patients meeting CDC criteria and 29 age-/sex-matched controls underwent standardized NFC at 200× magnification by a blinded examiner. Eight fingers (excluding thumbs) were imaged. Morphological parameters (tortuosity, crossing, dilatation, neoangiogenesis, etc.) and quantitative measures (capillary density, lengths, widths, intercapillary distance) were recorded. Tortuosity and crossing were scored semi-quantitatively (0: absent; 1: < 50% affected; 2: > 50% affected). Between-group comparisons used Mann–Whitney U and Chi-square/Fisher tests (*α* = 0.05).

**Results:**

MIS-C patients had higher rates of tortuosity (92.0% vs. 62.1%, *p* = 0.010), crossing (64.0% vs. 27.6%, *p* = 0.007) and dilated capillaries (16.0% vs. 0%, *p* = 0.040). Apical loop width was reduced (median 12 vs. 14 µm, *p* < 0.001) and disorganization score increased (*p* = 0.020). Semi-quantitative scores were higher for tortuosity (*p* = 0.002), crossing (*p* = 0.003) and dilatation (*p* = 0.027) in MIS-C. No meandering, giant capillaries, avascular areas or microhemorrhages were observed.

**Conclusion:**

Nailfold capillaroscopy revealed notable microvascular alterations in children with MIS-C, including increased tortuosity, dilated capillaries, and disorganization. These findings suggest that NFC may serve as a useful non-invasive tool for detecting endothelial dysfunction and early microvascular involvement in MIS-C. Importantly, given the current rarity of MIS-C cases in the post-pandemic era, this study provides a unique and timely contribution, offering one of the few systematic evaluations of microvascular alterations in pediatric MIS-C and establishing a valuable reference point for future comparative research in pediatric vasculopathies.

## Introduction

Multisystem Inflammatory Syndrome in Children is a severe hyperinflammatory condition that typically develops within 2–6 weeks following SARS-CoV-2 infection in childhood. This condition is characterized by persistent fever, elevated inflammatory markers, and multiorgan involvement, and it bears clinical resemblance to Kawasaki disease and toxic shock syndrome (1, 2). Although the global incidence of MIS-C has markedly declined with widespread vaccination and the emergence of newer SARS-CoV-2 variants, its potential for severe outcomes continues to warrant clinical attention.

The clinical spectrum of MIS-C is quite broad, and identifying severe cases at an early stage remains challenging. In a retrospective study conducted by Çetin et al. involving 99 MIS-C patients, certain admission biomarkers were shown to be associated with more severe clinical forms (3). Although its exact pathophysiology has not yet been fully elucidated, endothelial dysfunction and the resulting microvascular damage are thought to play a critical role in the disease process (4).

SARS-CoV-2 primarily enters host cells via the angiotensin-converting enzyme 2 (ACE2) and transmembrane serine protease 2 (TMPRSS2) receptors (5). Following viral entry, systemic immune activation is triggered, during which levels of endothelial activation markers such as von Willebrand factor (vWF) and P-selectin, along with proinflammatory cytokines including IL-6, IL-1β, and TNF-α, are elevated. This inflammatory response contributes to the development of thromboinflammation and endothelial damage (6–9). Autopsy studies conducted in the advanced stages of the disease have revealed marked endothelial inflammation and injury, suggesting that endotheliitis may play a central role in COVID-19-associated microvascular dysfunction (10).

Nailfold capillaroscopy is a non-invasive and practical method that enables high-resolution, real-time visualization of the peripheral microcirculation. This technique allows for the direct assessment of capillary structures that run parallel to the skin surface (11). NFC has long been used in rheumatology practice for the diagnosis and monitoring of connective tissue diseases. It is particularly useful in the diagnosis of systemic sclerosis, enabling the detection of characteristic findings such as capillary dilatation, tortuosity, architectural disorganization, and neoangiogenesis (12). These capillaroscopic findings have been incorporated into the 2013 classification criteria for systemic sclerosis established by the American College of Rheumatology (ACR) and the European League Against Rheumatism (EULAR) (13). Furthermore, studies have shown that endothelial damage markers may be elevated in individuals with Raynaud's phenomenon even before morphological abnormalities become detectable, highlighting the potential of NFC in identifying early endothelial injury (14).

Although studies on cardiovascular and systemic inflammatory findings associated with MIS-C are increasingly being conducted, microvascular involvement has not yet been sufficiently investigated. The ability of NFC to detect early microvascular changes makes it a promising tool for examining peripheral microcirculatory disturbances in MIS-C. Furthermore, the identification of such capillaroscopic abnormalities may also serve as an indirect indicator of endothelial dysfunction or disease severity (15).

Therefore, the aim of our study is to evaluate nailfold capillaroscopy findings in pediatric patients diagnosed with MIS-C and to determine the presence and extent of microvascular alterations associated with MIS-C by comparing these findings with those of age- and sex-matched healthy controls.

## Materials and methods

This cross-sectional, observational study was conducted in the Pediatric Rheumatology Unit of Erciyes University Faculty of Medicine between August and December 2021.The study included pediatric patients who met the Centers for Disease Control and Prevention (CDC) diagnostic criteria for MIS-C. Age- and sex-matched healthy peers without any known acute, chronic or systemic illness were recruited as the control group. The treatment of patients was conducted in accordance with the clinical management protocol developed by Erciyes University for MIS-C (16). Capillaroscopic evaluations were performed either during the discharge preparation period following treatment or within the first week after discharge during the initial follow-up visit. Written informed consent was obtained from all participants' legal guardians, and the study was approved by the local ethics committee (Decision No: 2022/115; Date: 09.02.2022), in accordance with the Declaration of Helsinki and Good Clinical Practice guidelines.

### Eligibility criteria

Patients with a confirmed diagnosis of MIS-C who were aged between 3 and 18 years were included in the study. The lower age limit of 3 years was selected due to the technical challenges and lower reliability of nailfold capillaroscopy in younger children. Exclusion criteria for both groups included a prior diagnosis of rheumatologic or autoimmune disease. In the control group, children with a history of hand trauma, known chronic systemic disease, or exposure to vasoactive medications (such as calcium channel blockers, beta-blockers, or systemic corticosteroids) were excluded. Additionally, control participants with recent febrile illness or signs of active infection at the time of evaluation were not enrolled, to minimize potential microvascular confounding factors. Hand dominance was documented for capillaroscopic standardization purposes: one patient in the MIS-C group and two individuals in the control group were left-hand dominant, while all other participants were right-hand dominant.

### Nailfold capillaroscopy protocol

All subjects underwent a 20-minute acclimatization period in a temperature-controlled room (20–24°C) prior to NFC imaging (17). NFC was conducted using a video microscope (MEDL4N Dino-Lite Pro Capillary Scope, Dino-Lite Europe, NN Almere, The Netherlands) at 200× magnification. A drop of immersion oil was applied to the nailfold to enhance image clarity. Eight fingers of both hands (excluding thumbs) were assessed for each participant, with special attention paid to the ring finger of the non-dominant hand. From each finger, two images were captured, resulting in 16 images per subject. All evaluations were performed by a single pediatric rheumatologist with three years of experience in capillaroscopy, who was blinded to the clinical status of the subjects.

Morphological abnormalities assessed included capillary tortuosity, crossing, dilatation, giant capillaries, meandering, ramification, microhemorrhages, pericapillary edema, avascular areas, and neoangiogenesis (e.g., bushy or pathologically branched capillaries). In accordance with previously described semi-quantitative scoring systems, ramifications were defined as branching, bushy, or coiled capillaries, often originating from a single normal-sized capillary (11, 18). Capillaries were defined as dilated when their internal diameter exceeded 20 µm and as giant when ≥50 µm, in accordance with international consensus definitions (18). This approach is also consistent with the definitions adopted in previous pediatric studies (19, 20). Avascular zones were defined as areas with intercapillary distances exceeding 500 μm in the distal row (21). Capillary meandering was defined by the repeated crossing of limbs over themselves or with adjacent loops (22). Tortuosity and capillary crossing were scored semi-quantitatively as 0 (absent), 1 (<50% capillaries affected), or 2 (>50% affected), with scores ≥2 considered significant (23). Architectural disorganization, along with capillary length, arterial and venous limb widths, total capillary width, and intercapillary distance, were considered exploratory variables outside the standardized EULAR definitions for nailfold capillaroscopy, but were included to provide additional descriptive information (18).

Measurements for capillary length, apical loop width, arterial and venous limb widths, and intercapillary distances were taken from three consecutive capillaries in the ring finger of the non-dominant hand (21). Quantitative parameters were calculated per patient by averaging the values obtained from the examined fingers. In cases where image quality was insufficient or a capillary loop could not be clearly visualized, such measurements were excluded from the analysis, and the average of the remaining measurable capillaries for that patient was used. Additionally, the Microangiopathy Evaluation Score (MES), as proposed by Sulli et al., was calculated for each subject (19). MES provides a semi-quantitative assessment of capillary loss, architectural disorganization, and presence of ramified capillaries, with each domain scored between 0 and 3, yielding a total score ranging from 0 to 9. In addition to the EULAR-standardized parameters (capillary density, apical width, abnormal morphologies, and microhemorrhages), exploratory variables such as capillary length, arterial and venous limb widths, total capillary width, intercapillary distance, and architectural disorganization were also recorded to provide complementary descriptive information (18).

### Statistical analysis

Descriptive statistics were expressed as mean ± standard deviation or median (min–max) for continuous variables, and as frequencies (*n*) and percentages (%) for categorical variables. The Shapiro–Wilk test was used to evaluate the normality of data distribution. The Mann–Whitney U test was used for comparisons of continuous variables between patient and control groups. For comparisons of nominal variables across groups (in contingency tables), the Chi-square test or Fisher's exact test was applied, as appropriate. Given the exploratory design and small sample size of the study, no formal correction for multiple comparisons (e.g., Bonferroni or FDR) was applied in order to avoid inflating type II error. All statistical analyses were conducted using IBM SPSS Statistics version 20 (Chicago, IL, USA), and a *p*-value < 0.05 was considered statistically significant.

## Results

### Demographic and baseline characteristics

A total of 54 children were enrolled in the study, comprising 25 patients with a history of MIS-C (10 females, 15 males) and 29 age- and sex-matched healthy controls (12 females, 17 males). The two groups did not differ significantly in median age [7 years (3–15) vs. 7 years (4–15), *p* = 0.951) or sex distribution (*p* = 0.918) (Table 1).

**Table 1:** Demographic characteristics of MIS-C patients and healthy controls.

Variable	MIS-C (*n* = 25)	Control (*n* = 29)	*p*-value
Age (years), median (min–max)	7 (3–15)	7 (4–15)	0.951^a^
Sex, *n* (%)			0.918^b^
Female	10 (40.0%)	12 (41.4%)	
Male	15 (60.0%)	17 (58.6%)	

Data are presented as median (min–max) for continuous variables and as number (%) for categorical variables.

^a^ Mann–Whitney U test,

^b^ Chi-square test (or Fisher's exact test, as appropriate).

The clinical characteristics of MIS-C patients are summarized in Table 2. The majority of patients presented with fever, rash, and gastrointestinal symptoms, while shock and respiratory involvement were less common. Cardiac evaluation most frequently revealed valvular regurgitation and bradycardia; the median ejection fraction was 66% (45–81), generally within the normal range, and no coronary artery involvement was observed. Laboratory findings were characterized by markedly elevated inflammatory and cardiac biomarkers, including CRP, ferritin, D-dimer, and NT-proBNP. All patients received corticosteroids, the majority were treated with IVIG and aspirin, and approximately half received anticoagulation therapy.

**Table 2:** Clinical, cardiac, laboratory, and treatment characteristics of children with MIS-C.

Characteristic	MIS-C patients (*n* = 25)
Demographic data	
Age (years), median (min–max)	7 (3–15)
Sex, *n* (%)	Female 10 (40.0%), Male 15 (60.0%)
Duration of fever before admission (days), median (min–max)	5 (1–10)
Length of hospital stay (days), median (min–max)	7 (4–19)
Intensive care unit admission, *n* (%)	3 (12.0%)
Clinical findings	
Fever, *n* (%)	25 (100.0%)
Shock/Hypotension, *n* (%)	2 (8.0%)
Gastrointestinal symptoms (abdominal pain/diarrhea/vomiting), *n* (%)	21 (84.0%)
Rash, *n* (%)	22 (88.0%)
Respiratory symptoms (dyspnea/cough), *n* (%)	7 (28.0%)
Conjunctivitis, *n* (%)	19 (76.0%)
Strawberry tongue, lip erythema/fissuring), *n* (%)	14 (56.0%)
Periorbital edema, *n* (%)	8 (32.0%)
Extremity swelling/edema or palm/sole changes, *n* (%)	8 (32.0%)
Lymphadenopathy, *n* (%)	2 (8.0%)
Cardiac findings	
Myocarditis, *n* (%)	0 (0.0%)
Ejection fraction (%), median (min–max)	66 (45–81)
Valvular regurgitation, *n* (%)	19 (76.0%)
Coronary artery involvement (increased echogenicity/dilatation/aneurysm), *n* (%)	0 (0.0%)
Bradycardia during follow-up, *n* (%)	14 (56.0%)
Pulmonary imaging findings	
Normal, *n* (%)	21 (84.0%)
Abnormal, *n* (%)	4 (16.0%)
Laboratory parameters at admission	
CRP (mg/L), median [IQR]	120.1 [66.5–183.3]
Ferritin (ng/mL), median [IQR]	546 [455–893]
D-dimer (µg/mL FEU), median [IQR]	5,910 [3,500–11,650]
Fibrinogen (mg/dL), median [IQR]	410.5 [311.8–620.3]
Troponin (ng/mL), median [IQR]	9.8 [0.01–38.3]
NT-proBNP (pg/mL), median [IQR]	4,877 [2,124–10,769]
WBC (/mm^3^), median [IQR]	5,760 [4,350–7,530]
Lymphocytes (/mm^3^), median [IQR]	1,020 [710–1,160]
Neutrophils (/mm^3^), median [IQR]	3,590 [2,320–6,540]
Platelets (/mm^3^), median [IQR]	124,000 [95,000–219,000]
Hemoglobin (g/dL), median [IQR]	9.8 [9.4–10.8]
Treatment	
IVIG, *n* (%)	22 (88.0%)
Steroids, *n* (%)	25 (100.0%)
Biologic therapy (anakinra), *n* (%)	0 (0.0%)
Aspirin, *n* (%)	23 (92.0%)
Anticoagulant therapy (enoxaparin), *n* (%)	13 (52.0%)

Data are presented as median (min–max) or median [IQR] for continuous variables, and as number (%) for categorical variables. BNP, B-type natriuretic peptide; CRP, C-reactive protein; EF, ejection fraction; MIS-C, multisystem inflammatory syndrome in children; NT-proBNP, N-terminal pro-B-type natriuretic peptide; WBC, white blood cell.

### Morphological NFC findings

According to the EULAR consensus definitions, abnormal capillary morphology was observed in 7 MIS-C patients (28.0%), whereas all healthy controls exhibited only normal morphologies (*p* = 0.011) (11, 18). The most frequent abnormalities in the MIS-C group included capillary dilatation (16.0%), triple crossing (16.0%), bushy capillaries (12.0%), and neoangiogenesis (12.0%) (Table 3). In line with the EULAR consensus, capillaries with an internal diameter >20 µm were classified as dilated and considered among abnormal morphologies; however, this feature may represent a relatively non-specific finding and should be interpreted with caution (Table 3).

**Table 3:** Capillary morphology in MIS-C patients and healthy controls.

Capillary morphology	MIS-C (*n* = 25)	Controls (*n* = 29)	*p*-value
Normal	18 (72.0%)	29 (100.0%)	0.011^a^
Abnormal (any)	7 (28.0%)	0 (0.0%)	
- Dilatation	4 (16.0%)	0 (0.0%)	
- Bushy (ramification)	3 (12.0%)	0 (0.0%)	
- Neoangiogenesis	3 (12.0%)	0 (0.0%)	
- Triple crossing	4 (16.0%)	0 (0.0%)	

Data are presented as *n* (%). Abnormal capillary morphology was defined according to the EULAR consensus as the presence of dilatation (>20 µm), giant capillaries (≥50 µm), ramification (bushy/neoangiogenesis), bizarre morphology, avascular areas, microhemorrhages, or triple crossing. While dilatation was included among abnormal morphologies based on consensus criteria, it should be noted that this finding may also represent a non-specific vascular change.

^a^ Chi-square or Fisher's exact test, as appropriate.

### Consolidated analysis of quantitative, morphological, and semi-quantitative NFC parameters

All quantitative, morphological, and semi-quantitative capillaroscopic parameters are summarized in Table 4. The MIS-C group had a significantly lower apical loop width compared to controls [median 12 µm (9–17) vs. 14 µm (11–17), *p* < 0.001] and significantly higher disorganization scores (*p* = 0.020). Although differences in density, capillary length, arterial and venous limb widths, capillary width, and intercapillary distance did not reach statistical significance, a trend toward higher density and capillary length was noted in MIS-C patients.

**Table 4:** Consolidated summary of nailfold capillaroscopic parameters in MIS-C patients vs. healthy controls.

Parameter	MIS-C (*n* = 25)	Control (*n* = 29)	*p*-value
A. Quantitative measures median (min–max)			
Density (/mm)	9 (7–10)	8 (7–10)	0.055^a^
Capillary length (µm)	254 (147–452)	216 (133–321)	0.096^a^
Arterial limb width (µm)	10 (7–14)	10 (8–12)	0.105^a^
Venous limb width (µm)	13 (11–19)	13 (11–15)	0.119^a^
Apical loop width (µm)	12 (9–17)	14 (11–17)	**<0**.**001**^a^
Capillary width (µm)	36 (29–52)	38 (26–48)	0.378^a^
Intercapillary distance (µm)	112 (87–135)	102 (80–145)	0.400^a^
Disorganization score	0.00 (0–0.38)	0.00 (0–0.25)	**0**.**020**^a^
B. Morphological findings (binary)			
Increased tortuosity	23 (92.0%)	18 (62.1%)	**0**.**010**^b^
Capillary tortuosity	None: 2 (8.0%) < 50%: 6 (24.0%) > 50%: 17 (68.0%)	None: 11 (37.9%) < 50%: 14 (48.3%) > 50%: 4 (13.8%)	**<0**.**001**^b^
Increased crossing	16 (64.0%)	8 (27.6%)	**0**.**007**^b^
Capillary crossing	None: 9 (36.0%) < 50%: 11 (44.0%) > 50%: 5 (20.0%)	None: 21 (72.4%) < 50%: 8 (27.6%) > 50%: 0 (0.0%)	**0**.**005**^b^
Dilated capillaries	4 (16.0%)	0 (0.0%)	**0**.**040**^b^
Giant capillary	0 (0.0%)	0 (0.0%)	**–**
Avascular area	0 (0.0%)	0 (0.0%)	**–**
Capillary meandering	0 (0.0%)	0 (0.0%)	**–**
Bushy capillaries	3 (12.0%)	0 (0.0%)	**0**.**093**^b^
Neoangiogenesis	3 (12.0%)	0 (0.0%)	**0**.**093**^b^
Microhemorrhages	0 (0.0%)	0 (0.0%)	**–**
C. Semi-quantitative scores			
Tortuosity score	0.62 (0.00–1.0)	0.25 (0.00–0.88)	**0**.**002**^a^
Crossing score	0.25 (0.00–0.88)	0.00 (0.00–0.50)	**0**.**003**^a^
Dilated capillary score	0.00 (0.00–0.50)	0.00 (0.00–0.00)	**0**.**027**^a^
Meandering score	0.00 (0.00–0.00)	0.00 (0.00–0.00)	**–**
Bushy capillary score	0.00 (0.00–0.50)	0.00 (0.00–0.00)	**0**.**057**^a^
Neoangiogenesis score	0.00 (0.00–0.50)	0.00 (0.00–0.00)	**0**.**057**^a^

Continuous variables are presented as median (min–max); categorical variables as *n* (%).

Bold values indicate statistically significant results (*p* < 0.05).

^a^ Mann–Whitney U test;

^b^ Chi-square or Fisher's exact test, as appropriate. In addition to EULAR-standardized parameters (capillary density, apical width, abnormal morphology, and microhemorrhages), exploratory quantitative variables such as capillary length, arterial and venous limb width, capillary width, intercapillary distance, and disorganization score were also recorded, which are not included in the standardized EULAR definitions.

Morphological analysis revealed that abnormalities were significantly more prevalent in MIS-C patients (Table 4). Increased tortuosity was observed in 92.0% of MIS-C patients vs. 62.1% of controls (*p* = 0.010), with severe tortuosity (>50% of capillaries affected) present in 68.0% of MIS-C cases compared to 13.8% in controls (*p* < 0.001). Crossing abnormalities were also more frequent in MIS-C patients (64.0% vs. 27.6%, *p* = 0.007), with severe crossing (>50%) observed in 20.0% of MIS-C patients but absent in controls (*p* = 0.005).

Dilated capillaries were significantly more common in the MIS-C group (16.0% vs. 0%, *p* = 0.040). Bushy capillaries and neoangiogenesis were detected in a minority of MIS-C patients (12.0% each) and absent in controls; although these differences did not achieve statistical significance (*p* = 0.093), they may indicate early angiogenic changes. No cases of giant capillaries, avascular areas, capillary meandering, or microhemorrhages were identified in either group. MES was zero for all participants.

As presented in Table 4, median tortuosity scores were significantly higher in MIS-C patients compared to controls [0.62 (0.00–1.00) vs. 0.25 (0.00–0.88), *p* = 0.002]. Crossing scores were also elevated [0.25 (0.00–0.88) vs. 0.00 (0.00–0.50), *p* = 0.003], as were dilated capillary scores (*p* = 0.027). Scores for meandering, bushy capillaries, and neoangiogenesis were zero in all participants, with no statistically significant differences between the groups.

**Figure 1:**
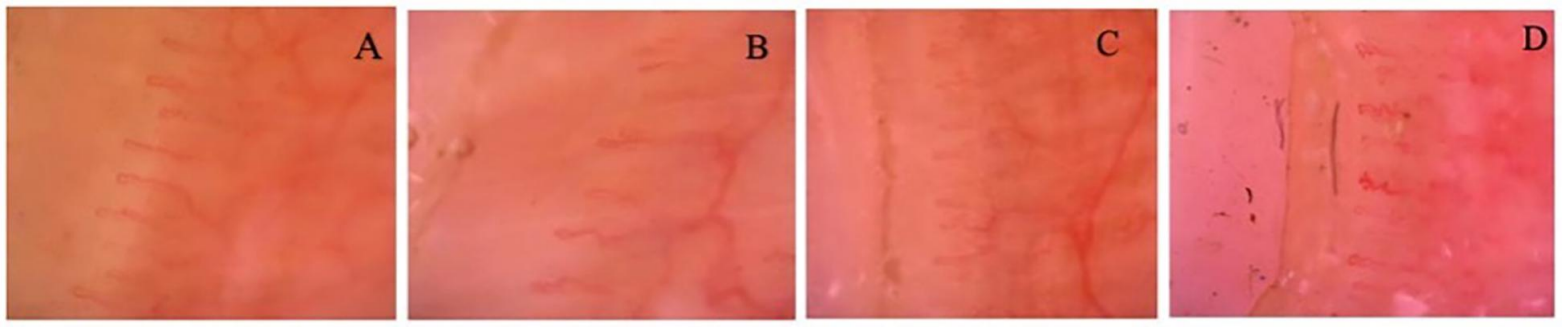
**(A)** Healthy control (10-year-old male): regularly arranged, parallel capillary loops with preserved morphology and uniform density. No abnormalities such as tortuosity, hemorrhages, giant capillaries, or neoangiogenesis are observed. **(B)** MIS-C* patient (15-year-old male): Prominent capillary tortuosity and mild architectural disorganization are evident. **(C)** MIS-C* patient (7-year-old male): Marked tortuosity with elongated, irregular loops and early branching, suggestive of incipient neoangiogenesis. **(D)** MIS-C* patient (4-year-old male): Severe tortuosity and mild disorganization without giant capillaries, hemorrhages, or neoangiogenesis. Capillary density is preserved. All images captured at 200× magnification using video capillaroscopy. *Multisystem Inflammatory Syndrome in Children.

Representative nailfold capillaroscopy images from both groups are shown in Figure 1, illustrating typical tortuosity, crossing, and dilated capillary patterns in MIS-C patients compared with the predominantly normal morphology in controls.

## Discussion

This study demonstrated significant morphological differences in microvascular structures assessed by nailfold capillaroscopy between children in the early post-acute phase of MIS-C and age- and sex-matched healthy controls. Specifically, marked increases in capillary tortuosity, capillary crossing, and frequency of dilated capillaries, along with a reduction in apical loop width, were observed in the MIS-C group. In addition, when applying the EULAR consensus binary classification, abnormal capillary morphology was identified in 28% of MIS-C patients, whereas no abnormalities were detected among healthy controls (18). This standardized framework further supports the robustness of our findings by highlighting a clear distinction between patients and controls. It should also be noted that architectural disorganization, although recorded in our study, is not part of the formally standardized NVC parameters according to the EULAR consensus, and thus these results should be interpreted with caution (18). These findings suggest that MIS-C may lead to endothelial dysfunction and disruption of peripheral microcirculatory structural integrity during the early recovery period.

Although data on microvascular dysfunction in children diagnosed with MIS-C secondary to COVID-19 remain limited, endothelial dysfunction is increasingly recognized as a central component in the pathophysiology of MIS-C (24–26). In a systematic review by Ciortea et al., significantly elevated levels of von Willebrand factor (vWF) and soluble E-selectin (sE-selectin) were reported in patients with MIS-C, highlighting their association with endothelial activation and microvascular inflammation. The same study also emphasized that cardiac involvement resulting from endothelial dysfunction may serve as an important prognostic indicator (27). Vascular dysfunction observed in MIS-C is believed to result from the hyperinflammatory response triggered by SARS-CoV-2 infection. Varga et al. (2020) identified COVID-19–associated endotheliitis in multiple organs, demonstrating its potential to cause widespread microvascular injury (10). These findings suggest that the systemic hyperinflammatory response observed in MIS-C may be associated with widespread endothelial activation and microvascular alterations.

In our study, laboratory parameters were routinely obtained from all patients. However, given the clinical characteristics of MIS-C, performing nailfold capillaroscopy during the acute phase was not feasible. The potential for severe disease course, the high risk of transmission requiring strict protective measures, and the prolonged and technically demanding nature of the procedure in children collectively limited the feasibility of capillaroscopic assessment in this phase. Consequently, the examinations were conducted only in the early post-acute period, during which laboratory parameters were generally within normal or near-normal ranges. Since the primary objective of our study was to determine whether microvascular involvement could be detected in the early post-acute phase of MIS-C through capillaroscopic evaluation, laboratory data were not additionally reported. This approach allowed capillaroscopic findings to be interpreted as independent observations, thereby providing a unique contribution regarding the presence of microvascular alterations in this population.

Beyond the immediate vascular alterations, the presence of subclinical microcirculatory changes in our MIS-C cohort highlights that the disease process may extend beyond the acute inflammatory period. Such findings are clinically important because endothelial injury and microvascular remodeling are increasingly recognized as contributors to long-term cardiovascular morbidity in children recovering from MIS-C. Therefore, NFC could be considered not only a diagnostic adjunct but also a potential monitoring tool to identify patients at risk of persistent vascular sequelae.

Smith et al. (2023) highlighted that NFC is a valuable tool for detecting early vascular dysfunction and that such morphological alterations may be observed even before the clinical onset of systemic diseases (11). In a capillaroscopic analysis conducted by Çakmak et al. (2021) in children diagnosed with COVID-19 and MIS-C, various microvascular abnormalities were reported, including capillary branching, coiling, tortuosity, ramification, bushy capillaries, microhemorrhages, and neoangiogenesis (15). Similarly, Tamez-Rivera documented pronounced capillary irregularities and structural alterations in a pediatric case of MIS-C, directly demonstrating microvascular involvement specific to the condition (28). Natalello et al. (2021) emphasized that findings such as capillary dilatation, tortuosity, and reduced capillary density observed by NFC in adult COVID-19 patients may represent direct indicators of endothelial injury (29). In this context, the increased frequency of capillary tortuosity, crossing, and dilated capillaries observed in our MIS-C cohort supports the reliability of NFC as a valuable tool for assessing the impact of systemic inflammation on microvascular structures.

In the study by Çakmak et al., which included 31 children with a history of SARS-CoV-2 infection (25 with COVID-19 and 6 with MIS-C) and 58 healthy controls, the frequencies of capillary ramification, tortuosity, microhemorrhages, neoangiogenesis, and coiling were significantly higher in the COVID-19 group compared to healthy individuals. Additionally, the patient group exhibited significantly lower capillary density and length, along with increased intercapillary distance. Morphologically, capillary ramification, crossing, dilatation, and neoangiogenesis were more frequently observed in MIS-C patients compared to those who had only experienced COVID-19 (15). In our study as well, microvascular structures assessed by NFC were significantly more impaired in the MIS-C group compared to healthy controls. In particular, increased tortuosity, capillary crossing, and the presence of dilated capillaries (defined as >20 µm according to international consensus) suggest subclinical endothelial injury or microvascular involvement secondary to inflammatory processes. Although the increases in ramification and neoangiogenesis did not reach statistical significance, a clear trend was observed. These findings indicate that the morphological changes have not yet progressed to advanced structural damage but are detectable at a subclinical level through NFC. This further supports the utility of NFC as a sensitive tool for detecting early microvascular alterations.

Recent findings in juvenile dermatomyositis (jDM) have highlighted the diagnostic and potential prognostic utility of nailfold capillaroscopy (NFC). In a recent single-center study, Bica et al. demonstrated that reduced capillary density and the presence of giant capillaries were significantly associated with skin disease activity and the presence of specific myositis-specific autoantibodies, particularly anti-TIF1*γ*. These results underscore the clinical relevance of microvascular abnormalities in pediatric systemic autoimmune diseases and support the growing role of NFC as a non-invasive biomarker for disease monitoring and patient stratification (30). Similarly, Barth et al. (2018) reported that persistent NFC abnormalities during long-term follow-up in jDM reflect irreversible microvascular damage (20). The absence of late-stage findings such as giant capillaries or avascular areas in MIS-C patients in our study suggests that the observed microvascular alterations may be reversible. In a prospective case-control study conducted by Boever et al. (2024), 17 MIS-C patients and 17 healthy controls were evaluated both during the acute phase and again at a median of 114.5 days (IQR: 94.5–136.0 days) using Sidestream Dark Field (SDF) imaging to assess sublingual microcirculation. Significant differences were found in total vessel density, microvascular flow index, proportion of perfused vessels, and vessel diameter distribution. Parameters measured with the EndoPAT device—such as the reactive hyperemia index and augmentation index, which reflect endothelial dysfunction and arterial stiffness—also supported the presence of endothelial impairment. While pronounced microcirculatory disturbances were observed in MIS-C patients during the acute phase, partial improvement in these parameters was reported at 3–4 months of follow-up. However, the authors emphasized that microvascular recovery was incomplete and that long-term cardiovascular outcomes should not be overlooked (31). Taken together, these findings support the hypothesis that NFC could provide valuable prognostic information in MIS-C. Detecting early and potentially reversible microvascular changes may help distinguish children who will recover completely from those at risk of persistent endothelial dysfunction, thereby informing follow-up strategies and therapeutic decisions.

Studies investigating the relationship between NFC findings and pediatric inflammatory diseases suggest that this non-invasive technique holds broader potential for evaluating inflammatory conditions in children (32). Moreover, similar capillaroscopic alterations have been described in other childhood vasculitides such as Kawasaki disease. In a case-control study conducted by Sedaghat et al., which included 31 patients diagnosed with Kawasaki disease and 30 healthy controls, a significantly higher frequency of capillary abnormalities was observed in the KD group (33). This suggests that the vascular effects observed in MIS-C may not be disease-specific but rather associated with the intensity of the inflammatory response.

This study has several limitations. First, it was conducted in a single center with a relatively small number of participants, which may limit the generalizability of the findings. Additionally, although our study was cross-sectional, this design inherently precludes the temporal evaluation of nailfold capillaroscopy findings. We did not perform an *a priori* sample size calculation because case availability during the study period was unpredictable and our primary objective was exploratory—to determine whether any microvascular involvement could be captured by NFC in the early post-acute phase of MIS-C. In this context, reliable assumptions for effect size and variance were not available. Instead, we report effect size estimates with 95% confidence intervals for key outcomes and consider our findings hypothesis-generating, warranting confirmation in larger, multicenter cohorts. In addition, multiple comparisons were conducted without formal adjustment for type I error. Given the exploratory design and small sample size, statistical corrections such as Bonferroni or FDR were not applied in order to avoid inflating type II error. Therefore, the results should be interpreted with caution and considered hypothesis-generating. A further limitation of our study is the absence of endothelial biomarker data (such as vWF, endothelin, sE-selectin, and VEGF), which could have provided additional insight into the mechanisms of endothelial dysfunction in MIS-C. However, these markers were not part of routine clinical management during the study period, and patient safety considerations in the acute phase limited the feasibility of obtaining additional biological samples. Given the end of the pandemic and the current rarity of MIS-C cases, conducting new prospective studies incorporating such biomarkers has become increasingly difficult. Therefore, our study adds valuable information to the limited literature obtained during the pandemic era. Despite the current rarity of MIS-C cases, future longitudinal and multicenter studies are needed to assess the progression of microvascular changes over time and their potential association with clinical outcomes in this population.

Given the decreasing incidence of MIS-C and the effective control of the COVID-19 pandemic, investigating such clinical presentations in the future is likely to become increasingly challenging. Therefore, data obtained through NFC in the acute phase not only help fill a significant gap in the current literature but also enhance the unique value of our study. Indeed, studies directly demonstrating MIS-C-related microvascular alterations through NFC remain extremely limited. In this context, our study offers a meaningful contribution to the existing body of knowledge and reinforces the scientific basis for microvascular assessment in this distinctive patient population.

## Conclusion

This study demonstrated that in children diagnosed with MIS-C, significant microvascular alterations can be detected through NFC performed in the early post-flare period, thereby providing one of the few pediatric data sets in the literature. Specifically, the presence of increased capillary tortuosity, structural disorganization, and capillary dilatation strongly suggests the impact of systemic hyperinflammation on peripheral microcirculation and the potential presence of endothelial dysfunction. The ability to reliably identify such early microvascular changes using NFC is particularly clinically relevant.

As a non-invasive, safe, and easily applicable method, NFC emerges as a valuable diagnostic tool for assessing microvascular involvement in children with MIS-C. Beyond its diagnostic utility, our findings also indicate that NFC may serve as a prognostic biomarker to distinguish children at risk of persistent vascular alterations. Importantly, in the post-pandemic era, MIS-C cases have become exceedingly rare, rendering the conduct of similar prospective studies virtually impossible. This rarity underscores the unique value of our work, positioning it as one of the few systematic and pediatric-focused contributions exploring microvascular involvement in MIS-C. Our findings provide significant insights into the understanding of systemic vascular dysfunction in this condition and may serve as a reference point for future comparative research on pediatric inflammatory and vasculopathic diseases.

## Data Availability

The original contributions presented in the study are included in the article/Supplementary Material, further inquiries can be directed to the corresponding author.
